# Synergic Effect of Selected Ingredients and Calcium Chloride on the Technological, Molecular and Microbial Usefulness of Eggshells and Their Impact on Sensory Properties in a Food Model System

**DOI:** 10.3390/ijms22042029

**Published:** 2021-02-18

**Authors:** Krystyna Szymandera-Buszka, Joanna Kobus-Cisowska, Daria Szymanowska-Powałowska, Ryszard Rezler, Oskar Szczepaniak, Grzegorz Marciniak, Justyna Piechocka, Maciej Jarzębski

**Affiliations:** 1 Department of Gastronomy Sciences and Functional Foods, Poznan University of Life Sciences, ul. Wojska Polskiego 28, 60-637 Poznań, Poland; krystyna.szymandera_buszka@up.poznan.pl (K.S.-B.); oskar.szczepaniak@up.poznan.pl (O.S.); justyna.piechocka@up.poznan.pl (J.P.); 2 Department of Biotechnology and Food Microbiology, Poznan University of Life Sciences, ul. Wojska Polskiego 28, 60-637 Poznań, Poland; darszy@up.poznan.pl; 3 Department of Pharmacognosy, Poznań University of Medical Sciences, ul. Heliodora Święcickiego 4, 60-781 Poznań, Poland; 4 Department of Physics and Biophysics, Poznan University of Life Sciences, ul. Wojska Polskiego 38/42, 60-637 Poznań, Poland; ryszard.rezler@up.poznan.pl (R.R.); maciej.jarzebski@up.poznan.pl (M.J.); 5 Department of Microeconomics and Food Economics, Poznan University of Economics and Business, al. Niepodległości 10, 61-875 Poznań, Poland; grzegorz.marciniak@ue.poznan.pl

**Keywords:** calcium, eggshells, inulin, lactose, lysine, magnesium, X-ray diffraction

## Abstract

Lower levels of calcium in adults increase the risk for osteoporosis, and in children, low calcium levels can impact their potential adult height. The study objective was to analyze the bioavailability and physicochemical properties of a calcium preparation based on chicken eggs. The base calcium preparation was enriched with one of a variety of biologically active substances, inter alia, vitamin D3, vitamin K, lysine, lactose, magnesium chloride and inulin. The newly developed calcium preparations were subjected to structural analysis using X-ray diffraction and scanning electron microscopy, and the hydrodynamic diameter for the molecules was determined using the dynamic light scattering method and their zeta potential. To determine the optimum storage conditions of calcium preparations, their hygroscopicity and bulk density were determined. The calcium preparations were also added to selected food products, such as apple juice with mango, fruit dessert (jelly) and beef meatballs. The enriched food products were subjected to sensory analysis. The study demonstrated the significant influence of additives to calcium preparation in terms of its hygroscopicity and morphology. It was found that all products with the addition of analyzed preparations were characterized by high sensory desirability. The results presented in the study comprise the basis for the development of new food products, enriched with calcium.

## 1. Introduction

It has long been known that a healthy, balanced diet consisting of wholesome products has a positive and highly significant impact on human health. However, it should be emphasized that even in highly developed countries, the issue of improper organism nutrition exists, which is a consequence of way of life, eating in fast-food restaurants, using diets without consulting a dietitian, and lack of knowledge of proper nutrition. Calcium is one of the most important micronutrients of the organism. The total content of calcium in the organism is about 1200 mg, which is 1.5% of the weight of an adult person [1]. The calcium resources in the bones and teeth are estimated at 99%, and support their structure and function. A total of 1% of the total pool of calcium present in the organism is present in the blood serum. Calcium content in the blood serum is strictly regulated. Maintaining a constant calcium concentration in the blood, intercellular fluids and muscles is possible thanks to the capacity for its storage in bone tissue [2]. The best method for supplementing calcium deficiency in the organism is its provision with food. However, in many cases, this is not sufficient; thus, it is increasingly common to seek alternative solutions aiming at supplementation of such deficiencies. One example is calcium preparations in the form of supplements or food enriched with calcium salts. Chicken eggshells constitute a very good, natural source of calcium. They contain 94 g calcium carbonate, including 40 g of calcium per 100 g of raw material [3,4,5]. As an example, chicken egg contains 1.33 g calcium/100 g, and in a rennet whey cheese (Edam fat), the calcium concentration is equal to 8.67 g/100 g. However, vegetable material is characterized by low calcium content, e.g., apples (0.04 g/100 g), or bananas (0.06 g/100 g). Meat is also a poor source of calcium (pork—0.18 g/100 g, poultry—0.10 g/100 g), and furthermore, the considerable amounts of phosphorus and saturated fatty acids further inhibit its absorption. It should be remarked that apart from calcium, an eggshell contains over 500 different proteins. Such proteins contain specific matrices binding metal ions, and which can be freely released from them. Thus, apart from calcium, an eggshell is a valuable source of magnesium, zinc, iron, fluorine, silica and molybdenum. Important components included in eggshell are magnesium carbonate, phosphates, glycoproteins, polysaccharides, amino acids and hormones [6,7,8]. In comparison with other natural sources of calcium carbonate, eggshells are also characterized by elevated content of not only calcium, but also strontium and selenium, and lower content of such harmful metals as vanadium, lead, cadmium chromium and aluminum [9,10,11].

One of the more important issues associated with consumption of calcium-rich foods is its bioavailability. However, substances exist that contribute to the increased absorption of calcium in the gastrointestinal tract, thus considerably increasing its bioavailability and also biofunctionality [12,13,14]. Such compounds include vitamins, mostly vitamin D3 and vitamin K2, and elements such as magnesium, sugars such as lactose and inulin, as well as amino acids, for instance lysine [15,16,17,18]. It should be noted that literature data state that calcium carbonate obtained from eggshells is characterized by higher bioavailability compared to synthetic products due to the specific chemical composition and porous structure, increasing its solubility and favoring a more efficient release of calcium ions in the stomach [19].

The use of roasted, milled eggshells is known in the specialized literature [20,21]. Considering the proven health-promoting properties of chicken eggshell, obtaining high-protein preparations as biofunctional food additives is a highly interesting and technologically attractive solution.

The objective of this study was the development, physiochemical assessment, description of the molecular structure and implication for food products of an innovative preparation containing easily absorbable calcium obtained from chicken eggs, additionally enriched with one bioactive substances such as vitamin D3, vitamin K, lysine, lactose, magnesium chloride or inulin.

## 2. Results and Discussion

During the study, eggshell preparations were tested containing different additives: inulin GR (EGR), inulin HSI (EHSI), lysine (ELI), lactose, vitamin D3 (ED), MgCl_2_ (ECM), vitamin K (EK) and control sample with no additive (EC).

### 2.1. Analysis of Calcium Preparation Structure

#### 2.1.1. Analysis of Morphology and Structure of Calcium Preparations

**Figure 1:**
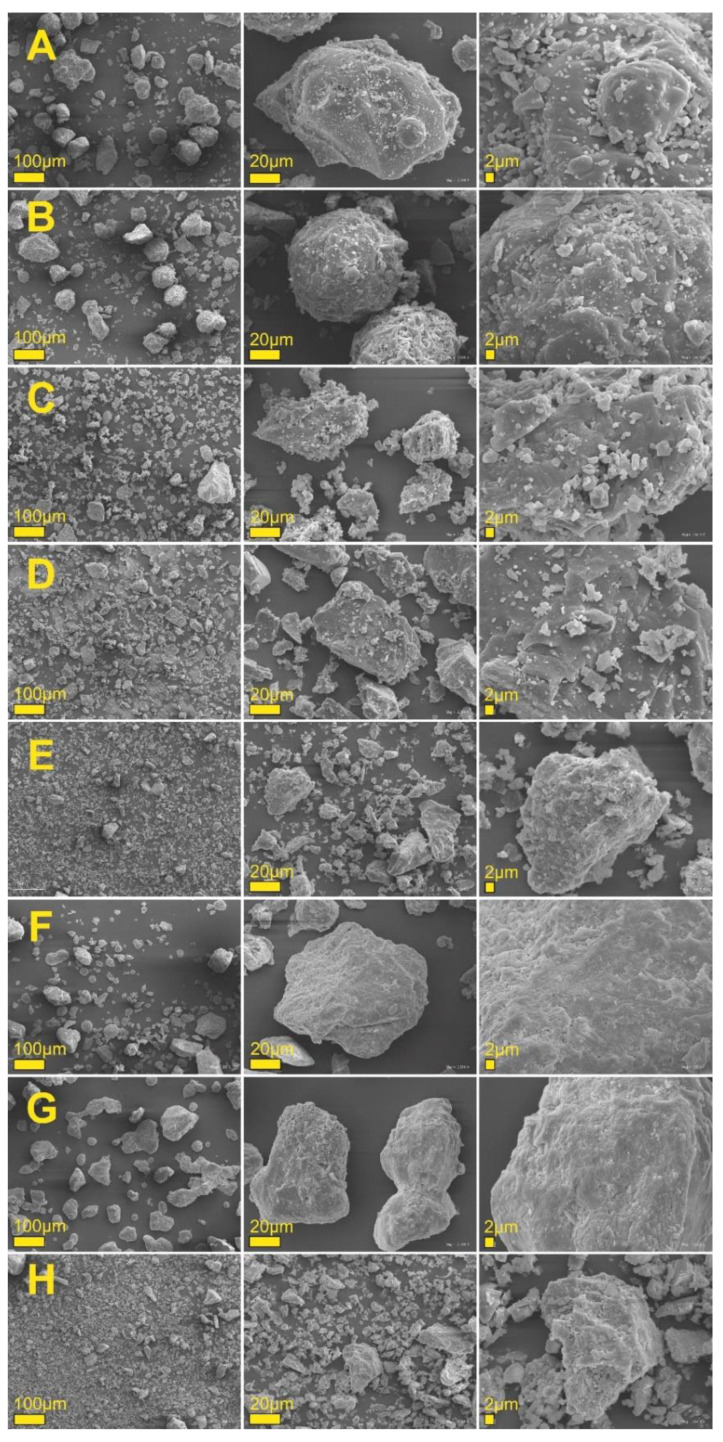
SEM images of the studied calcium matrices. (**A**) EGR, (**B**) EHSI, (**C**) ELI, (**D**) ELA, (**E**) ED, (**F**) ECM, (**G**) EK, (**H**) EC.

**Figure 2:**
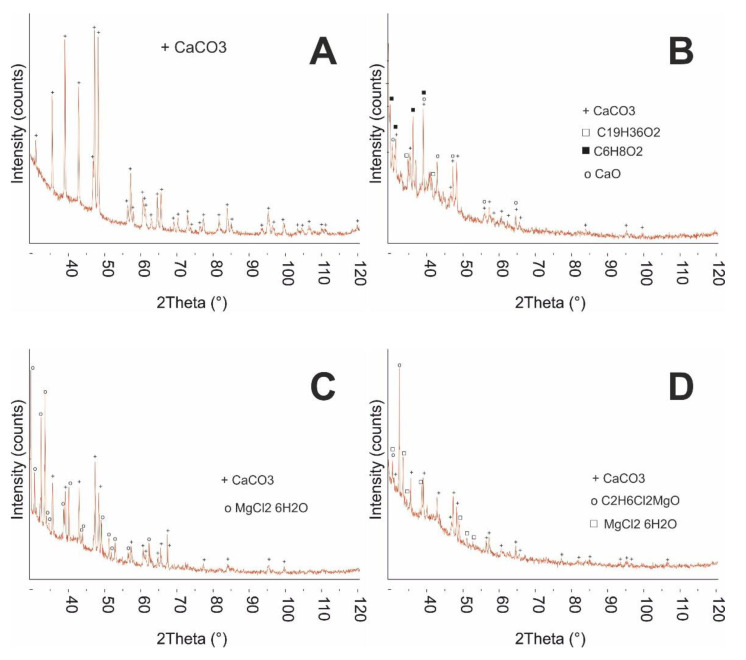
XRD diffractograms of studied calcium matrices. (**A**) EC, (**B**) ELI, (**C**) ECM, (**D**) EK.

Microscopic studies greatly increase the evaluation process of food product homogeneity [22]. Possible agglomerates or other inhomogeneous structures in the final product might impact sensory evaluation [23,24]. The morphology and structure of the newly developed calcium preparations were examined using scanning electron microscope (SEM) and X-ray diffraction, and the distributions of the sizes of hydrodynamic radii of particles in water were determined. Figure 1 presents SEM images of the baseline and modified preparations, recorded at different magnifications. Both the baseline preparation (Figure 1H) and the modified samples (Figure 1A–G) were characterized by heterogeneous crystalline structures with the shape of irregular granules. Figure 1F and 1G are missing single particles, and only show large foci of particles of crystalline nature, as well as particles with a size of several nm. Undoubtedly, the morphology of calcium powders was influenced by the additives introduced to them. To perform a more in-depth structure analysis, the powder chemical composition analysis was examined via X-ray diffraction. Figure 2 presents diffractograms of the selected prepared powders. Figure S1 shows diffractograms of other eggshell powders. Calcium carbonate is a predominant compound.

In the mixture variants containing inulin (EGR and EHSI), peaks were identified that also originate from calcium carbonate. In the mixture containing lysine (EL), apart from chicken eggshell, peaks originating from both calcium carbonate, as well as calcium oxide and organic structures, were identified (at this stage, no further analyses of organic compound structure were performed—additional XRD diffractograms are presented in the Supplementary Materials). In turn, in the mixture containing lactose, apart from the peak originating from calcium carbonate, clear peaks originating from calcium chloride and organic compounds were found. Analysis of a diffractogram of the mixture containing chicken eggshell and vitamin D3 did not unambiguously demonstrate whether a magnesium carbonate was formed in addition to calcium carbonate. The ECM mixture consisted of calcium carbonate as well as hydrated magnesium chloride, which translated into a different morphology of the powder, as presented in the figure (Figure 1). The most complex crystallographic structure was found for the preparation containing, apart from chicken eggshell, also the addition of vitamin K. The presence of hydrated magnesium chloride could be associated with the elevated hygroscopicity of the tested preparations. This translates into the need to use special protections against moisture and adaptation of the packaging and storage method against possible caking and formation of particle agglomerates.

#### 2.1.2. Determination of Hydrodynamic Diameter and Zeta Potential of Calcium Preparation Particles

**Figure 3:**
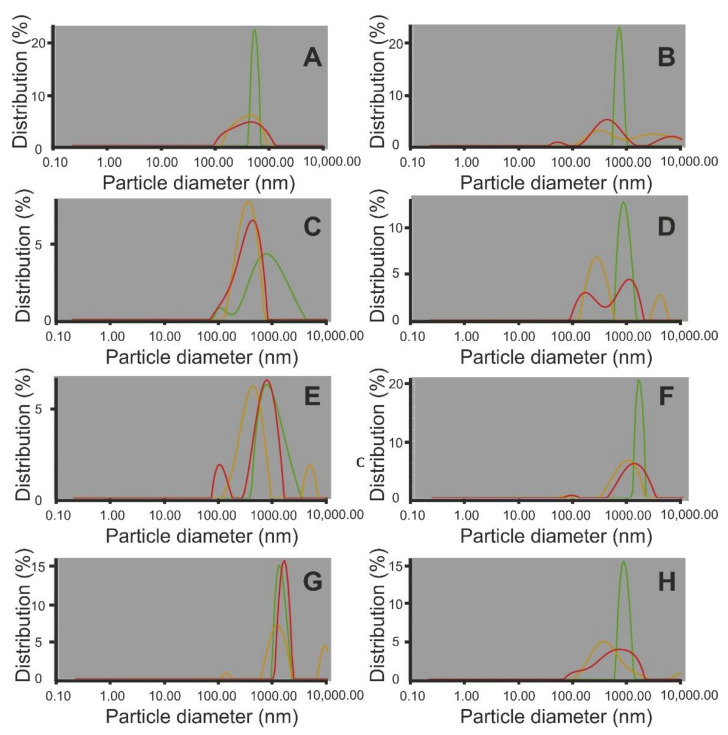
Particle size distribution in the tested calcium matrices. Green series—measured at an angle of 15°, yellow series—measured at an angle of 90°, red series—measured at an angle of 175°). (**A**) EGR, (**B**) EHSI, (**C**) ELI, (**D**) ELA, (**E**) ED, (**F**) ECM, (**G**) EK, (**H**) EC.

Dynamic light scattering (DLS) is one of the most commonly used techniques for the rapid analysis of particle size distribution, including nanoparticles in foods [25]. DLS can be successfully applied for food nanoemulsion stability studies [26,27,28]. It should be noted that in highly polydispersed samples, such as those investigated, the intensity of the scattered light from even a few large particles (i.e., contamination, aggregates, agglomerates, larger) may strongly affect the average particle diameter [29]. However, the hydrodynamic radius is not correlated with the particle sizes determined using, i.e., electron microscopy. In the case of the studied calcium preparations, the DLS analysis only covered the supernatant originating from the particle suspension from water. The visible fractions that underwent sedimentation (aggregates identified via SEM) were not included in the above analysis. What is more, the autocorrelation functions originating from light dispersed by particles were recorded at three different angles (15, 90 and 175°). The results are compared in Table 1, and the graphic distribution of particle size is shown in Figure 3. The zeta potential was measured, which describes the surface properties of solids dispersed in non-polar or polar solvents and the interaction between colloidal particles. DLS measurements (Table 1) demonstrated that all prepared samples were characterized by a high degree of polydispersion, independent of the measurement angle (d15, d90, d175). At 15°, PDI ranged between 8 and 39, at 90°, it remained in the range 18–33, and at 175°, it was within 16–29. Figure 2 presents the graph for particle distribution, i.e., the percentage share of particles depending on their size. The main peak had a very wide range, translating into a high PDI index. In all powder samples, fractions of several hundred nanometers or several micrometers were identifiable. The presented study results confirm the analyses performed using SEM imaging. The zeta potential in all samples ranged between −18 and −24 mV. On this basis, it can be observed that stability was not found in any of the tested models

### 2.2. Analysis of Physicochemical Properties of the Produced Preparations

The acidity analysis (Table 2) of the developed preparations demonstrated the highest value of this indicator (9.50–9.36) for the preparations with the addition of inulin and vitamin D3. The statistically significantly different sample with the lowest level of this indicator (7.58) was the preparation with the addition of lysine. Another analyzed marker was bulk density, which indicates the significant relationship associated with the packing of the particle deposits, that is, compressibility. This marker makes it possible to characterize the applicability of the powdered material for further marketing and transport, storage and processing [30]. Based on the obtained results, it was determined that the lowest bulk density (0.43 g/cm^3^) was exhibited by the control variant, consisting solely of the eggshell (EGR). The additives used resulted in its increase. The addition of lysine had the lowest impact on the increase of this marker. On the other hand, the greatest bulk density (1.07 g/cm^3^) was found for the samples containing vitamin D3, as well as the chicken eggshells (ED). It was noted that the amount or quality of ingredients included in the given preparation did not influence its bulk density. The analysis of ash content (Table 2) demonstrated statistically significant differences resulting from the composition of the introduced additives. The highest content of mineral compounds was found for the mixture with addition of magnesium chloride (71%). A content lower by approximately 34% was found in preparations with the addition of inulin and lactose, which should be associated with the composition of the added ingredient. The remaining preparations were characterized by similar ash content, at the level of approximately 50%.

### 2.3. Microbiological Analysis

Microbiological tests of semi-finished products constitute an important stage in the production of food additives, supplements and food, because they determine their safety. By testing the microbiological purity of the prepared mixtures, the total count of mesophilic and psychrophilic microorganisms, the total counts of yeasts and mesophilic and psychrophilic molds, as well as the counts of acidifying bacteria were determined, testing the presence of *Salmonella, Listeria* and *Enterobacteriaceae.* All preparations were characterized by microbiological purity. Bacteria with pathogenic potential were not determined in any of the variants.

### 2.4. Results of the Assessment of Sensory Quality of Food Products with the Addition of High-Protein Biofunctional Products

**Figure 4:**
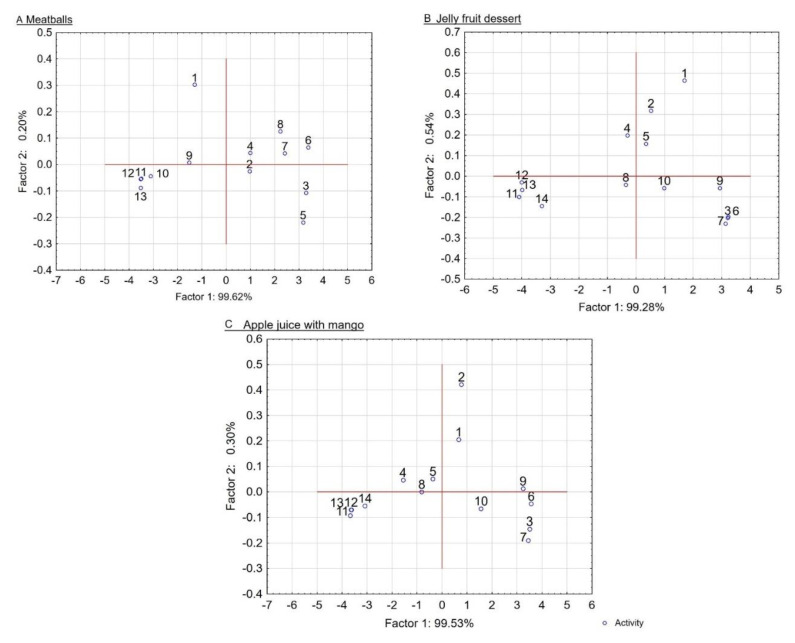
The projection of cases on the factor plane for samples coding for (**A**) Meatballs: 1—meat taste 2—salty taste, 3—bitter taste, 4—fatty taste, 5—powdered taste, 6—metallic taste, 7—red color, 8—gray color, 9—brown color, 10—uniformity of color, 11—taste desirability, 12—color desirability, 13—general desirability; (**B**) Jelly fruit dessert; and (**C**) Apple juice with mango: 1— sour taste, 2—sweet taste, 3—bitter taste, 4—fruit taste, 5—mango taste, 6—powdered taste, 7—metallic taste, 8—yellow color, 9—brown color, 10—clarity of color, 11—uniformity of color, 12—taste desirability, 13—color desirability, 14—general desirability.

The last stage of the conducted work was to apply the developed preparations in three food products: a beverage, a dessert and a meat dish. The prepared products were subjected to consumer assessment, and the obtained results are presented in Table 3 and in Figure 4. Based on the obtained results, it was determined that all products with the addition of analyzed preparations were characterized by similar and high sensory desirability, independently of the variant of preparation added.

Through the sensory analysis, it was found that all products with the addition of analyzed preparations were characterized by similar and high sensory desirability, independently of the variant of preparation added. Earlier studies showed that the application of eggshell to fried cheese makes the breakfast product a valuable source of bioavailable calcium and is a sensory attractive alternative to dietary supplements [22]. Szeleszczuk et al. [31] observed nearly 100% dissolution of calcium from eggshell after a 90-min digestion process with only an 80% ratio for pure CaCO_3_. In our previous study [22], calcium supplement based on eggshells resulted in over 23% simulated bioavailability, while when applied with vitamins D and K in lipid food matrix, the calcium availability increased by approximately 57%.

No adverse effects on the sensory properties of these products were noticed. The consumer evaluation results showed that the biscuit and steamed cakes with and without the addition of eggshells were characterized by similar and high levels of desirability [32]. The results indicated a higher yellow color intensity.

The highest total desirability was observed in the meatball assessment. Consumers declared high desirability for both the taste and color of all product variants. The general desirability of the products was rated in the range between 8.9 and 9.2 points on the 10-point scale. The highest levels of total desirability and taste desirability were determined for samples containing the eggshell preparation without additives, but this was not statistically significant. In the sensory profile assessment of the meatball samples, perception of the following descriptors was defined and determined: color (red, brown, gray, uniformity), and taste (meat, salty, bitter, fat, powder, metallic). Based on the analysis of the obtained profile testing results (PCA), one principal component was distinguished (Figure 4), corresponding to 99% of variance of sensory quality of the tested meatball samples. Independently of the introduced preparation variant, very similar color and taste profilograms were determined. No statistically significant differences were found between color intensity for all present sensations (Table 3). All variables were characterized by a similar high uniformity of color (8.5 cm) equality on the surface and on the cross-section. In all samples, the highest intensity was found for brown color (ranging from 6.3 to 6.6 cm), with lower intensities being found for gray (1.3–1.8 cm) and red (1.2–1.5 cm).

Similar tendencies were found in the profile assessment of taste. All samples were characterized by similar intensities of salty (3.0–3.4 cm) and, fatty (3.0–3.2 cm) and powdered tastes. Similar intensities were found in the profile assessment of bitter (0.0 cm) and powdered (0.0–0.3 cm). Significant factors include the perception of powdered and metallic tastes. Earlier studies have shown that bitter taste can decrease consumer acceptance of food products, particularly those for which this taste is not characteristic [33,34,35]. Additionally, metallic taste is an important problem in food technology, for example, with sweeteners.

Significant deviations in comparable profilograms were determined only for the meat taste (*p* < 0.05) in the samples with the addition of the calcium preparation with magnesium chloride additives (ECM). In the ECM samples, an increase of bitter taste intensity (from 0.0 to 1.0) and powdered (from 0.1 from 1.5) was observed. Additionally, significant deviations for intensity of taste were determined for the meat taste descriptors (*p* < 0.05). In the samples with the addition of calcium preparation without additives (EC), an increase in meat taste intensity was observed, from 5.8 to 7.5.

Principal component analysis (PCA) was used to study the relations between the taste and color attributes characteristic for the sensory profiles of meatballs (variables) and to derive factors according to which these variables can be classified. The PCA showed that the first factor (F1) was the most important element for explaining variation in the data (over 99%). It is worth highlighting that F1 was strongly related to all taste and color attributes. A clear separation was found between groups of sensory characteristics on the PC biplot (Figure 4). Salty, bitter, fatty and powdered taste, and red and gray color were positioned to the right of PC1 and negatively correlated with meat taste, and brown and uniformity of color.

Similar tendencies were determined in the assessment of apple juice with the addition of mango, as well as the jelly-type dessert with mango.

Based on the obtained results, it was determined that all samples with the addition of the analyzed preparations were characterized by similar and high levels of sensory desirability, independently of the variant of preparation added. No significant differences were found in the consumer reception of any of the analyzed variables. Consumer assessment of all samples indicated high desirability for color and taste (7.2–9.1). The general desirability of the products was rated in the range between 8.0 and 8.6 points on the 10-point scale.

No significant differences were found in the consumer reception of any of the analyzed variables for either juice or the jelly-type dessert. In the assessment of the sensory profile of these products, the perception of the following descriptors was defined and determined: color (orange, yellow, creamy, white and clear), and taste (sweet, sour, bitter, fruity, mango, apple, powder, metallic). No statistically significant differences were found between color intensity for any of the present sensations (Table 3). All samples were characterized by similar high uniformity of color (9.0–9.3 cm). In all samples of dessert and juice, the highest intensities were found for yellow color (ranging from 4.5 to 4.7 cm and from 5.4 to 5.6 cm, respectively), and lower clarity (ranging from 2.8 to 3.2 cm and from 2.4 to 2.8 cm, respectively). Additionally, similar intensity was found in the profile assessment of brown color (0.4–0.7 cm).

Similar tendencies were found in the profile assessment of taste. All samples were characterized by similar intensity, responsible for 99% for the variability of sensory quality of the assessed jelly and 99% for juice. Independently of the variant of the preparation introduced, very similar color profilograms were determined. On the other hand, profile analysis of taste demonstrated statistically more intensive perception of sweet taste in the samples with preparation without additives and with lactose addition. This concerned samples of juice and jelly-type dessert. There was a stronger perception of fruit (dessert 4.2–5.2 cm and juice 6.0–7.1 cm), mango (dessert 3.4–4.2 cm and juice 4.8–5.4 cm) and powdered (from 0.0 to 0.7) taste.

Significant deviations in the intensity of taste were determined for the sour, sweet, bitter and metallic taste descriptors (*p* < 0.05). Similar intensities were found in the profile assessment for sour taste (1.5–2.2 cm) in the dessert samples but not for the samples with the addition of calcium preparation without additives (EC). In EC samples, an increase in sour taste intensity (from 2.0 to 3.7 cm) was found. In the samples with the addition of calcium preparation and lactose (ELA), an increase of sweet taste intensity (from 3.3 to 4.5) was observed.

In samples of jelly fruit dessert with ECM, an increase of bitter taste intensity (from 0.0–0.2 to 0.7 cm) and metallic taste (from 0.0–0.2 to 0.6 cm) were found. Higher intensity of bitter taste (0.7 cm) was also found for juice with ECM. In the juice samples with the addition of calcium preparation and lactose (ELA), an increase of sweet taste intensity (from 3.3 to 4.5) was observed. Additionally, in the samples with AC and EHSI, a higher intensity of sweet and sour taste descriptors was found.

Furthermore, reduction of the intensity of fruit and sweet taste was determined in the juice with preparation with lysine addition. However, these changes did not have an influence on the change of taste desirability and the total sensory desirability among consumers.

Analysis of the results obtained through profile testing (PCA) enabled the distinction of one principal component (Figure 4B,C), responsible for over 99% of the variability of sensory quality of the assessed jelly dessert and juice. It is worth highlighting that F1 was strongly related to all taste and color attributes. A clear separation of groups of sensory characteristics was found on the PC biplot (Figure 4) for samples of both apple juice and jelly dessert. Sour, sweet, mango, metallic, bitter and powdered taste, and brown and clarity of color of jelly fruit dessert were positioned to the right of PC1 and negatively correlated with fruit taste, and yellow and uniformity of color. For apple juice samples, sour, sweet, bitter, metallic and powdered taste, and clarity of color were positioned to the right of PC1 and negatively correlated with fruit and mango taste, and yellow and uniformity of color.

The results of our analysis confirmed the high intensity of yellow color for the dessert and juice samples. Our research also confirmed the high intensity of brown color for meatball samples. An increase was also found for yellow color intensity of breadcrumbs and brown intensity of crusts with increasing levels of eggshells. Brun et al. [19] confirmed the high desirability of pizza, bread and spaghetti with the addition of eggshell. The sensory evaluation also indicated the similarity of crispiness, flavor and taste profiles up to 10% levels of the same calcium source. On the other hand, when using higher levels, a stronger fishy smell was observed [36]. Our studies on dessert and juice samples confirmed the high intensity of sweet taste descriptors, especially for samples with inulin or lactose additives. Addition of inulin with short-chain molecules enhances flavor and sweetness, and can be used to partially replace sucrose [37].

## 3. Materials and Methods

### 3.1. Material

#### 3.1.1. Chemicals

The chemicals used in the study were purchased from chemical warehouses and producers in Poland, Germany and Lithuania. L-Lysine in the form of creamy-colored granules, devoid of taste and smell, was derived from NOW Foods, Bloomingdale, IL, USA. Vitamin K2 (Menaquinone-7, 0.1%) in the form of white powder, devoid of taste and smell, came from ForMeds Sp. z o.o., Poznań, Poland. According to the information obtained from the manufacturer, the vitamin was produced from geraniol and farnesol, with the addition of inulin from the chicory root. Vitamin D3 (1α, 25-dihydroxycholecalciferol) in the form of white powder, devoid of taste and smell, came from the ForMeds Sp.z o.o., Poznań, Poland. Granulated inulin (GR) and highly soluble inulin (HSI) in the form of white powder came from Hortimex Sp.z o.o., Konin, Polska. Lactose powder was purchased from AB Rokiskio suris, Rokiškis, Lithuania. Magnesium chloride granules were purchased from AGNEX, Białystok, Poland. Apple juice with mango was purchased from Hortex, Warsaw, Poland. Mango jelly was purchased from AGNEX, Białystok, Poland. Pork meat was purchased from a local store.

#### 3.1.2. Raw Materials

The raw materials were eggshells in powder form, creamy in color, with a slightly limestone smell and taste. The raw material was obtained from EGGNOVO SI, Navarra, Spain. The composition of the shell preparation was as follows: 98% calcium carbonate including 38% calcium, 2% soluble protein and 250 ppm strontium, pH 8.34, moisture 2.54, particle size 85% < 50 μm.

### 3.2. Sample Preparations

Compositions of seven preparations were developed, which were based on micronized chicken eggshell. The eighth preparation constituted the control, and contained micronized eggshell only. The quantitative and qualitative compositions of the developed preparations are presented in Table 4.

The obtained preparations were stored in PE/Al/PE bags. During packing, 80% of the air was removed in the chamber device and the bags were welded.

### 3.3. Structural Analyses

#### 3.3.1. X-ray Diffraction

X-ray diffraction was conducted with help of Panalytical Empyrean XRD X-ray diffractometer. A copper lamp (CuKα) was the source of X-ray radiation. The X-ray spectra were registered at angle range 2° (plane angle measure) between 30° and 120°. The Panalytical Empyrean XRD database (Malvern Panalytical, Malvern, UK) was used for the analysis of basic chemical composition.

#### 3.3.2. Scanning Electron Microscopy

Scanning electron microscopy (SEM) was used for the morphology test. The preparation samples were applied on adhesive carbon disks glued to the SEM observation tables. Subsequently, they were sprayed with gold for 4 min in an ionic sputter. The last step consisted of observation of the image in an electron microscope Evo40 (Carl Zeiss AG, Jena, Germany), at 12 kV voltage.

#### 3.3.3. Dynamic Light Scattering

Dynamic light scattering (DLS) was performed so as to determine the distribution of particle size. DLS was performed via Litesizer™ 500 by Anton Paar GmbH (Graz, Austria). Preparation of the test samples was identical to the zeta potential measurement. After collection of the supernatant over the sediment, measurement was performed in polystyrene cuvettes (10 × 10 × 45 mm), which were stabilized with suspension for 5 min in the DLS chamber. Automatic regulation of the laser beam was used for analysis, where: λ = 658 nm, laser strength 40 mW. Autocorrelation functions were registered for 15°, 90° and 175° angles, in order to increase the measurement sensitivity and estimate the possibility of detecting particles with lower diameters. Autocorrelation functions enable the determination of the particle diffusion coefficient in the solution (D), followed by determination of hydrodynamic radius (R_h_ or diameter d_h_) [24,38]. The relationship between D and R_h_ is provided by the Stokes-Einstein formula Equation (1):

(1) $$R_{h}=\frac{kT}{6\pi \eta D}$$

where: *k*—Boltzmann constant, *T*—temperature [K], *η*—solvent viscosity, *D*—diffusion coefficient.

#### 3.3.4. Zeta Potential

Zeta potential (ζ) measurement was performed via Litesizer™ 500 (Anton Paar GmbH, Graz, Austria). Several mg of calcium preparations were placed in 1 mL of deionized water; subsequently, the suspension was mixed using a vortex agitator for 1–2 min and additionally in an ultrasonic bath at temperature 20 °C for 15–30 min. Supernatant from the above sediment was collected for the analysis. The measurement was performed in polystyrene cuvettes (10 × 10 × 45 mm) of omega type, which were thermally stabilized with the suspension for 5 min in a measurement chamber. Zeta potential was recorded in the range −600 to +600 mV. The results were developed using Kalliope™ software (Anton Paar GmbH). The potential was determined on the basis of measurements electrophoretic mobility, after the transformation Henry’s formula Equation (2):

(2) $$\mu =\frac{2\varepsilon \varsigma f(Ka)}{3\eta }$$

where: *µ*—electrophoretic mobility, *ε*—dielectric constant, *f*(*Ka*)—Henry’s function, *η*—solvent viscosity.

### 3.4. Physicochemical Analyses

#### 3.4.1. Determination of Active Acidity

Determination of active acidity (pH) was performed via the electrometric method by measuring hydrogen ion activity using a PM-60C pH-meter with a readout accuracy of 0.01 pH. The measurement was performed in suspension prepared from 10 g of powder and 100 mL deionized water at 20 °C.

#### 3.4.2. Bulk Density

Bulk density was determined using a weight method. Electric analytical scales (Radwag, Poznań, Poland) were used to measure the weight of the prepared samples, whereas the volume of the weighed samples was measured with a graduated cylinder. The method consists of pouring the tested powder into a tared graduated cylinder to the final volume of 100 mL, and then measuring its mass on a balance. The entire procedure was done in triplicate for each sample. The obtained data were used to calculate the density of powder models, expressed in g/cm^3^.

#### 3.4.3. Ash Content

Ash content was determined using the weight method with the use of so-called ‘dry’ mineralization [39].

### 3.5. Microbiology

The microbiological quality of the tested parameters was assayed according to Polish Standards. The presence and number of aerobic mesophilic and psychrophilic microorganisms were determined according to the standard [40]. Molds and yeasts were assayed using the horizontal method. The growth medium was peptone dextrose (PD) agar with chloramphenicol (Biocorp, Warsaw, Poland). Incubation conditions were as follows: temperature 30 °C, aerobic conditions, and time 48–72 h. So as to determine the total number of bacteria of genus *Lactobacillus*, MRS-agar (Biocorp) was applied. Incubation temperature was 35 °C, and incubation time was 48 h [41]. Coli group bacteria were detected according to PN-ISO 4832:2007 [42], *Salmonella* subspecies were identified according to PN-EN ISO 6579-1:2017-04 [43], and *Enterobacteriaceae* genera according to PN-EN ISO 21528-2:2017-08 standard [44].

### 3.6. Implications of Chicken Eggshell Preparation for Food Products

The obtained preparations (EGR; EHSI; ELI; ELA; ED; ECM; EK; EC) at 2% (g/g d.m.) concentration were added to products such as apple juice with mango, jelly fruit dessert and beef meatballs. These products were also prepared without the addition of preparation (0E). In the case of apple and mango juice, the preparations were added to fluid, mixed and set aside for 15 min at a temperature of 4 °C. The jelly fruit dessert was prepared in accordance with the recipe provided by the producer, adding high-protein preparation to the dry product. Roasting meatballs-Pork (best end of the neck) was ground (mesh size of 3 mm) and mixed with the other ingredients, producing batter containing 70% meat, 15.8% water, 6% breadcrumbs, 5% eggs, 1% salt and 0.2% pepper and eggshell preparation. To maintain identical conditions determining the kinetics of thermal processing, samples of a similar weight (30 ±1 g) with a round shape were formed. Next, they were roasted (160 °C/20 min) in a convection oven (Rational, Landsberg am Lech, Germany).

### 3.7. Sensory Analysis of Products with Variants of Eggshell Preparations

Investigations were conducted in an appropriately designed and equipped laboratory for sensory analysis at the Department of Gastronomy Sciences and Functional Foods, Poznań University of Life Sciences, Poland. The sensory profiling was conducted by an eight-member trained panel. Simultaneously to profile analysis, consumer examination was conducted with the participation of 141 consumers, aged 22-32. Women constituted 54% of the population analyzed. Both consumer and profile analysis used a 10 cm hedonic graphic scale with appropriate margin descriptions. For attributes of color and taste for profile analysis, uniform margin denotations were applied: undetectable–very intensive. Meanwhile, for consumer analysis, these were undesirable–highly desirable.

### 3.8. Statistical Analysis

Principal Component Analysis (PCA) was used to perform the overall assessment of differences and similarities of sensory profiles of the tested products based on the color and distinguishing taste features. Results were subjected to one-way analysis of variance (Statistica 13.3, Statsoft, Cracow, Poland) using Tukey’s test. Hypotheses were tested at α = 0.05.

### 3.9. Safety Information

Preparations were made in a special food laboratory with all personal protective equipment provided (i.e., lab coat, gloves) so as not to contaminate the tested material. The mineralization was conducted in a chemical laboratory with the help of thermal gloves. The mineralization process was conducted in a furnace under a fume hood. Analyses involving X-rays were done in a special laboratory with the use of special protective covers. All growth media were prepared according to producer’s recommendations. After preparation, growth media were sterilized in an autoclave at a temperature of 121 °C for 20 min.

## 4. Conclusions

Calcium preparations produced based on chicken eggshells can constitute an alternative to the current calcium supplements. All analyzed preparations were characterized by heterogeneous crystalline structure with the shape of irregular granules. All preparation samples were characterized by high polydispersion level. The most complex crystallographic structure was exhibited by the calcium preparation with the addition of vitamin K. The conducted sensory tests for products enriched with the tested preparations demonstrated their high sensory desirability among consumers. The addition of the analyzed preparations may influence enhancement of the perception of the desired taste for the given product.

The introduced additives influenced morphology of calcium preparations. Furthermore, analysis of calcium preparations may effectively support the design of food products, at the same time being used to assess their safety. Further testing of new products enriched with calcium preparations based on chicken eggshells is recommended. All analyzed preparations were characterized by microbiological purity, heterogeneous crystalline structure with the shape of irregular granules and high polydispersion level. The most complex crystallographic structure was exhibited by the calcium preparation with the addition of vitamin K. The conducted sensory tests for products enriched with the tested preparations demonstrated their high sensory desirability among consumers. The addition of the analyzed preparations may influence enhancement of the perception of the desired taste for the given product.

The introduced additives influenced the morphology of calcium preparations. Furthermore, analysis of calcium preparations may effectively support the design of food products, while at the same time being used to assess their safety. Further testing of new products enriched with calcium preparations based on chicken eggshells is recommended.

## Figures and Tables

**Table 1:** Hydrodynamic diameter and zeta potential of modelled calcium powders.

MODEL	d_15_ (nm)	PDI (–)	d_90_ (nm)	PDI (–)	d_175_ (nm)	PDI (–)	ζ ± SD (mV)	EM (μm·cm·s^−^^1^·V^−^^1^)
EGR	917	32	370	28	372	24	21.0 ± 0.4	–16,352
EHSI	1053	31	698	37	1143	22	18.5 ± 0.8	–14,419
ELI	602	37	327	18	346	27	23.6 ± 0.4	–18,400
ELA	875	26	425	33	358	29	20.7 ± 0.5	–16,165
ED	785	18	451	27	677	27	20.5 ± 0.4	–15,971
ECM	2197	39	1049	27	1263	24	18.1 ± 0.4	–14,119
EK	2064	35	1983	33	2174	16	19.5 ± 0.3	–15,178
EC	934	22	501	27	752	26	19.1 ± 0.5	–14,897

Average of diameter of the population registered at the following angles: d_15_—15°, d_90_—90° and d_175_—175°. PDI—polydispersion coefficient, ζ—zeta potential, SD—standard deviation, EM—electrophoretic mobility.

**Table 2:** Physiochemical characteristics of tested preparations.

MODEL	pH	Bulk Density (g/cm^3^)	Ash (% Dry Matter)
EGR	9.36 ^ab^ ± 0.01	0.86 ^b^ ± 0.02	37.35 ^e^ ± 0.37
EHSI	9.50 ^a^ ± 0.14	0.90 ^b^ ± 0.01	34.20 ^f^ ± 0.12
ELI	7.58 ^f^ ± 0.01	0.58 ^e^ ± 0.01	51.55 ^a^ ± 0.81
ELA	9.04 ^d^ ± 0.01	0.70 ^d^ ± 0.01	34.30 ^f^ ± 0.17
ED	9.39 ^ab^ ± 0.01	1.07 ^a^ ± 0.03	51.80 ^a^ ± 0.40
ECM	8.75 ^e^ ± 0.07	0.86 ^b^ ± 0.05	71.90 ^b^ ± 0.75
EK	9.28 ^abd^ ± 0.02	0.73 ^cd^ ± 0.01	54.10 ^c^ ± 0.08
EC	8.34 ^bd^ ± 0.07	0.43 ^f^ ± 0.00	55.40 ^d^ ± 0.39

a–f—different letters in the same columns denote a significant difference at α ≤ 0.05 (n = 9).

**Table 3:** Characteristics of sensory taste profiling and consumer analysis of meatballs, jelly fruit dessert and apple juice with mango.

Variable	Factor–Variable Correlations (Factor Loadings), Based on Correlations						
	EC	EGR	EHSI	ELI	ELA	ED	ECM
Meatballs							
meat taste	7.5 *^b^*	5.0 *^a^*	5.5 *^a^*	6.0 *^a^*	6.2 *^a^*	6.0 *^a^*	6.1 *^a^*
salty taste	3.0 *^a^*	3.1 *^a^*	3.4 *^a^*	3.0 *^a^*	3.3 *^a^*	3.3 *^a^*	3.2 *^a^*
bitter taste	0.0 *^a^*	0.0 *^a^*	0.0 *^a^*	0.0 *^a^*	0.0 *^a^*	0.0 *^a^*	1.0 *^b^*
fatty taste	3.1 *^a^*	3.2 *^a^*	3.1 *^a^*	3.1 *^a^*	3.2 *^a^*	3.1 *^a^*	3.0 *^a^*
powdered taste	0.0 *^a^*	0.3 *^a^*	0.2 *^a^*	0.0 *^a^*	0.0 *^a^*	0.0 *^a^*	1.5 *^b^*
metallic taste	0.0 *^a^*	0.0 *^a^*	0.0 *^a^*	0.0 *^a^*	0.0 *^a^*	0.0 *^a^*	0.0 *^a^*
red color	1.2 *^a^*	1.3 *^a^*	1.2 *^a^*	1.2 *^a^*	1.5 *^a^*	1.2 *^a^*	1.3 *^a^*
gray color	1.6 *^a^*	1.6 *^a^*	1.3 *^a^*	1.8 *^a^*	1.6 *^a^*	1.5 *^a^*	1.3 *^a^*
brown color	6.5 *^a^*	6.4 *^a^*	6.4 *^a^*	6.6 *^a^*	6.4 *^a^*	6.3 *^a^*	6.4 *^a^*
uniformity of color	8.5 *^a^*	8.5 *^a^*	8.5 *^a^*	8.5 *^a^*	8.5 *^a^*	8.5 *^a^*	8.5 *^a^*
taste desirability	9.0 *^a^*	8.8 *^a^*	9.2 *^a^*	9.0 *^a^*	9.1 *^a^*	9.0 *^a^*	9.0 *^a^*
color desirability	9.0 ^a^	9.0 *^a^*	9.0 *^a^*	8.9 *^a^*	9.0 *^a^*	9.1 *^a^*	9.0 *^a^*
general desirability	9.0 *^a^*	9.0 *^a^*	9.0 *^a^*	8.9 *^a^*	9.0 *^a^*	9.0 *^a^*	9.2 *^a^*
Jelly fruit dessert							
sour taste	3.7 *^b^*	2.0 *^a^*	2.0 *^a^*	2.0 *^a^*	2.2 *^a^*	1.5 *^a^*	1.6 *^a^*
sweet taste	4.2 *^c,b^*	3.1 *^a^*	3.6 *^b,a^*	3.0 *^a^*	4.5 *^c,b^*	3.3 *^a^*	3.2 *^a^*
bitter taste	0.0 *^a^*	0.0 *^a^*	0.0 *^a^*	0.0 *^a^*	0.0 *^a^*	0.4 *^b^*	0.7 *^a^*
fruit taste	5.2 *^a^*	4.4 *^a^*	4.3 *^a^*	4.3 *^a^*	4.9 *^a^*	4.3 *^a^*	4.2 *^a^*
mango taste	4.2 *^a^*	3.5 *^a^*	3.4 *^a^*	3.5 *^a^*	4.3 *^a^*	3.6 *^a^*	3.6 *^a^*
powdered taste	0.0 *^a^*	0.0 *^a^*	0.0 *^a^*	0.0 *^a^*	0.0 *^a^*	0.0 *^a^*	0.7 *^a^*
metallic taste	0.0 *^a^*	0.0 *^a^*	0.0 *^a^*	0.5 *^b^*	0.0 *^a^*	0.5 *^b^*	0.5 *^b^*
yellow color	4.5 *^a^*	4.6 *^a^*	4.5 *^a^*	4.7 *^a^*	4.7 *^a^*	4.5 *^a^*	4.5 *^a^*
brown color	0.5 *^a^*	0.5 *^a^*	0.6 *^a^*	0.7 *^a^*	0.5 *^a^*	0.4 *^a^*	0.5 *^a^*
clarity of color	2.8 *^a^*	3.0 *^a^*	2.8 *^a^*	2.9 *^a^*	3.1 *^a^*	3.2 *^a^*	2.8 *^a^*
uniformity of color	9.0 *^a^*	9,2 *^a^*	9.0 *^a^*	9.1 *^a^*	9.2 *^a^*	9.2 *^a^*	9.3 *^a^*
taste desirability	9.0 *^a^*	9.0 *^a^*	9.2 *^a^*	9.1 *^a^*	9.1 *^a^*	9.0 *^a^*	8.9 *^a^*
color desirability	9.0 *^a^*	9.0 *^a^*	9.0 *^a^*	9.0 *^a^*	9.0 *^a^*	9.1 *^a^*	9.0 *^a^*
general desirability	8.0 *^a^*	8.2 *^a^*	8.2 *^a^*	8.0 *^a^*	8.0 *^a^*	8.3 *^a^*	8.4 *^a^*
Apple juice with mango							
sour taste	4.3 *^b^*	3.8 *^b,a^*	4.4 *^b^*	3.5 *^a^*	3.3 *^a^*	3.5 *^a^*	3.4 *^a^*
sweet taste	4.2 *^c,b^*	3.6 *^b,a^*	4.1 *^b^*	3.0 *^a^*	4.5 *^c^*	3.1 *^a^*	3.2 *^a^*
bitter taste	0.0 *^a^*	0.0 *^a^*	0.0 *^a^*	0.2 *^a^*	0.0 *^a^*	0.4 *^a^*	0.6 *^a^*
fruit taste	7.1 *^a^*	6.0 *^a^*	6.4 *^a^*	6.6 *^a^*	6.5 *^a^*	6.6 *^a^*	6.2 *^a^*
mango taste	5.4 *^a^*	5.0 *^a^*	5.0 *^a^*	4.8 *^a^*	4.9 *^a^*	5.1 *^a^*	4.8 *^a^*
powdered taste	0.0 *^a^*	0.0 *^a^*	0.2 *^a^*	0.0 *^a^*	0.0 *^a^*	0.0 *^a^*	0.4 *^a^*
metallic taste	0.0 *^a^*	0.0 *^a^*	0.0 *^a^*	0.5 *^b^*	0.0 *^a^*	0.5 *^b^*	0.5 *^b^*
yellow color	5.6 *^a^*	5.5 *^a^*	5.6 *^a^*	5.4 *^a^*	5.6 *^a^*	5.6 *^a^*	5.6 *^a^*
brown color	0.5 *^a^*	0.5 *^a^*	0.6 *^a^*	0.7 *^a^*	0.5 *^a^*	0.4 *^a^*	0.5 *^a^*
clarity of color	2.6 *^a^*	2.4 *^a^*	2.4 *^a^*	2.5 *^a^*	2.7 *^a^*	2.8 *^a^*	2.7 *^a^*
uniformity of color	9.0 *^a^*	9.2 *^a^*	9.0 *^a^*	9.1 *^a^*	9.0 *^a^*	9.2 *^a^*	9.0 *^a^*
taste desirability	9.0 *^a^*	9.0 *^a^*	9.0 *^a^*	9.1 *^a^*	9.1 *^a^*	9.0 *^a^*	9.2 *^a^*
color desirability	9.0 *^a^*	9.0 *^a^*	9.0 *^a^*	9.0 *^a^*	9.0 *^a^*	9.1 *^a^*	9.0 *^a^*
general desirability	8.5 *^a^*	8.3 *^a^*	8.2 *^a^*	8.2 *^a^*	8.4 *^a^*	8.3 *^a^*	8.6 *^a^*

Different lowercase *a*–*c* letters for particular products denote a significant difference between particular product enriched in tested preparations, at α ≤ 0.01 (one-way ANOVA at *p* < 0.01).

**Table 4:** Composition of the eggshell preparations.

	EGR	EHSI	ELI	ELA	ED	ECM	EK	EC
Ingredient	Content in preparation (%)							
Eggshell	35	35	35	35	87.5	50	96	100
Inulin GR	65	0	0	0	0	0	0	0
Inulin HSI	0	65	0	0	0	0	0	0
Lysine	0	0	65	0	0	0	0	0
Lactose	0	0	0	65	0	0	0	0
Vitamin D3	0	0	0	0	12.5	0	0	0
Magnesium chloride	0	0	0	0	0	50	0	0
Vitamin K	0	0	0	0	0	0	4	0

## Data Availability

The data presented in this study are available on request from the corresponding author.

## Supplementary Materials

The following are available online at https://www.mdpi.com/1422-0067/22/4/2029/s1, Figure S1. XRD diffractograms of studied calcium matrices. (A) EGR, (B) EHSI, (C) ELA, (D) ED.
