# Supplementary material for: Synergic Effect of Selected Ingredients and Calcium Chloride on the Technological, Molecular and Microbial Usefulness of Eggshells and Their Impact on Sensory Properties in a Food Model System
Source: Int J Mol Sci. 2021 Feb 18;22(4):2029. doi: 10.3390/ijms22042029 (PMC7922246; doi:10.3390/ijms22042029)
Supplement: Supplementary file 1 [file ijms-22-02029-s001.pdf]

Supporting materials

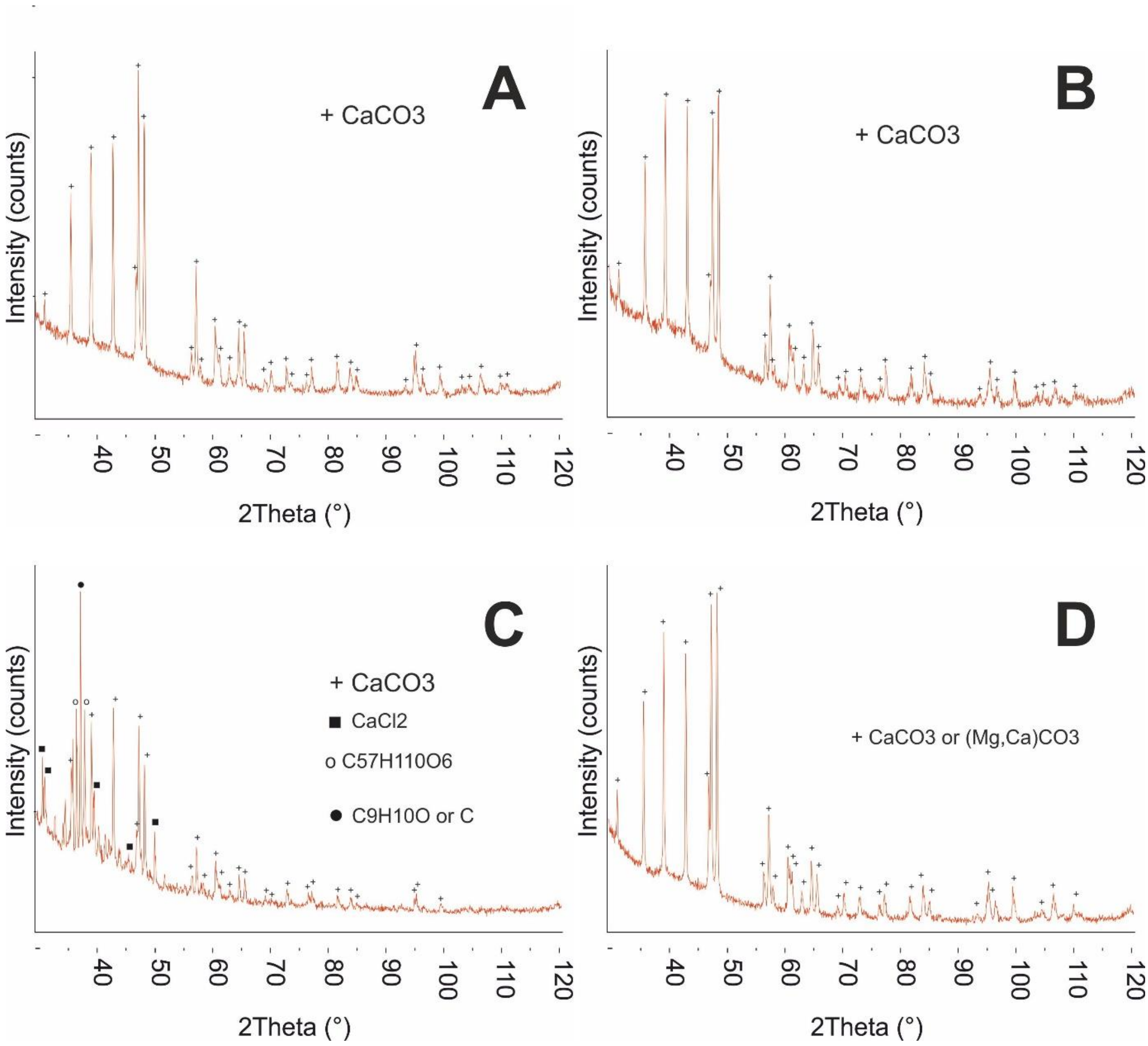

Figure S1. XRD diffractograms of studied calcium matrices. A-EGR, B- EHSI, C-ELA, D-ED.
